# Ecological Analyses of Mycobacteria in Showerhead Biofilms and Their Relevance to Human Health

**DOI:** 10.1128/mBio.01614-18

**Published:** 2018-10-30

**Authors:** Matthew J. Gebert, Manuel Delgado-Baquerizo, Angela M. Oliverio, Tara M. Webster, Lauren M. Nichols, Jennifer R. Honda, Edward D. Chan, Jennifer Adjemian, Robert R. Dunn, Noah Fierer

**Affiliations:** a Cooperative Institute for Research in Environmental Sciences, University of Colorado, Boulder, Colorado, USA; b Departamento de Biología y Geología, Física y Química Inorgánica, Escuela Superior de Ciencias Experimentales y Tecnología, Universidad Rey Juan Carlos, Móstoles, Spain; c Department of Ecology and Evolutionary Biology, University of Colorado, Boulder, Colorado, USA; d Department of Applied Ecology, North Carolina State University, Raleigh, North Carolina, USA; e Department of Biomedical Research, Center for Genes, Environment, and Health, National Jewish Health, Denver, Colorado, USA; f Department of Medicine, National Jewish Health, Denver, Colorado, USA; g Division of Pulmonary Sciences and Critical Care Medicine, University of Colorado Denver, Aurora, Colorado, USA; h Denver Veterans Affairs Medical Center, Denver, Colorado, USA; i National Institute of Allergy and Infectious Diseases, Bethesda, Maryland, USA; j United States Public Health Service Commissioned Corps, Rockville, Maryland, USA; k Natural History Museum of Denmark, University of Copenhagen, Copenhagen, Denmark; University of Georgia

**Keywords:** *Mycobacterium*, NTM lung disease, nontuberculous mycobacterial infection, plumbing biofilms

## Abstract

Bacteria within the genus *Mycobacterium* can be abundant in showerheads, and the inhalation of aerosolized mycobacteria while showering has been implicated as a mode of transmission in nontuberculous mycobacterial (NTM) lung infections. Despite their importance, the diversity, distributions, and environmental predictors of showerhead-associated mycobacteria remain largely unresolved. To address these knowledge gaps, we worked with citizen scientists to collect showerhead biofilm samples and associated water chemistry data from 656 households located across the United States and Europe. Our cultivation-independent analyses revealed that the genus *Mycobacterium* was consistently the most abundant genus of bacteria detected in residential showerheads, and yet mycobacterial diversity and abundances were highly variable. Mycobacteria were far more abundant, on average, in showerheads receiving municipal water than in those receiving well water and in U.S. households than in European households, patterns that are likely driven by differences in the use of chlorine disinfectants. Moreover, we found that water source, water chemistry, and household location also influenced the prevalence of specific mycobacterial lineages detected in showerheads. We identified geographic regions within the United States where showerheads have particularly high abundances of potentially pathogenic lineages of mycobacteria, and these “hot spots” generally overlapped those regions where NTM lung disease is most prevalent. Together, these results emphasize the public health relevance of mycobacteria in showerhead biofilms. They further demonstrate that mycobacterial distributions in showerhead biofilms are often predictable from household location and water chemistry, knowledge that advances our understanding of NTM transmission dynamics and the development of strategies to reduce exposures to these emerging pathogens.

## Abstract (precis)

Bacteria thrive in showerheads and throughout household water distribution systems. While most of these bacteria are innocuous, some are potential pathogens, including members of the genus *Mycobacterium* that can cause nontuberculous mycobacterial (NTM) lung infection, an increasing threat to public health. We found that showerheads in households across the United States and Europe often harbor abundant mycobacterial communities that vary in composition depending on geographic location, water chemistry, and water source, with households receiving water treated with chlorine disinfectants having particularly high abundances of certain mycobacteria. The regions in the United States where NTM lung infections are most common were the same regions where pathogenic mycobacteria were most prevalent in showerheads, highlighting the important role of showerheads in the transmission of NTM infections.

## INTRODUCTION

Bacteria grow and persist in biofilms coating the inside of showerheads and shower hoses despite the seemingly inhospitable conditions found in these habitats. These bacteria must tolerate rapid temperature fluctuations, long intervals of stagnation or desiccation followed by high-shear turbulent flow events, and the low nutrient and organic carbon concentrations typical of most drinking water. In many cases, showerhead-associated bacteria must also be able to tolerate residue from the chemical disinfectants (including chlorinated compounds) which are often added to municipal drinking water to limit bacterial contamination. Despite these stressors, bacterial abundances often exceed 10^6^ cells cm^−2^ inside shower plumbing (1–3). Thus, in the act of showering, we are exposed to elevated concentrations of showerhead-associated bacteria as they are dislodged and aerosolized (4–6).

Most of the bacteria that can become aerosolized and inhaled when the shower is in use are likely harmless. However, this is not always the case. Bacteria within the genus *Mycobacterium* are commonly detected in showerhead biofilms and throughout the water distribution system (7). There are nearly 200 described species of nontuberculous mycobacteria (NTM), which are defined as any members of the genus that are not M. tuberculosis or M. leprae—which cause tuberculosis and leprosy, respectively. Many species of NTM, including M. abscessus, M. kansasii, members of the M. avium complex (MAC) or M. fortuitum complex, and M. mucogenicum (8, 9), are frequently implicated as environmentally acquired pathogens that can cause an array of human diseases, most notably chronic suppurative lung disease; the skin and soft tissue infections often associated with surgical procedures or occupational exposures; cervical lymphadenitis in children; and disseminated infections among susceptible individuals, including those with compromised immune systems (10–12). Although NTM can be found in a range of environments, including soil, house dust, and natural bodies of water, exposure from showerheads may be more likely. The hydrophobic nature of NTM cells can enhance their potential for aerosolization from showerheads, and the inhalation of aerosolized NTM from showerheads is frequently implicated as an important route of NTM transmission (4, 13–15). Indeed, previous studies (5, 16–18) have shown that the mycobacterial strains recovered from NTM-infected patients can often be genetically matched to the mycobacterial strains found in the showers used by the patients.

NTM infections are increasingly recognized as a threat to public health as they are often difficult to treat and the prevalence of NTM lung disease is on the rise in the United States and other developed nations (7, 9, 19). Interestingly, the prevalences of NTM lung disease are not evenly distributed across geographic regions. For example, there are “hot spots” of NTM disease within the United States—in Hawaii, southern California, Florida, and the New York City area (20). Moreover, the specific NTM strains most often linked to respiratory infections may differ across geographic regions (19–22). However, it remains unclear why NTM lung disease is increasingly prevalent and why this geographic variation in NTM infections exists. We posit that these patterns are, in part, linked to differences in the amounts and lineages of mycobacteria found in showerheads.

As mycobacteria are significantly more resistant than other bacteria to chlorine and chlorine by-products (18, 23, 24), they are expected to be more abundant in showerheads and water distribution systems where such disinfectants are used. We also expect some mycobacteria to be more common in households receiving more-acidic water (18, 25, 26) and in water systems where free-living amoebae (FLA) are common, given that mycobacteria can survive and replicate inside amoebae (27–30). However, it remains unclear whether these abiotic and biotic variables can effectively predict the distributions of NTM in household water distribution systems and the likelihood of acquiring NTM opportunistic infections.

The diversity, distributions, and ecologies of those NTM colonizing showerheads are clearly relevant to public health. Unfortunately, we currently lack a comprehensive understanding of which mycobacteria are found in showerheads and how the distributions of mycobacterial taxa vary depending on geographic location, interactions with other microorganisms, and environmental conditions. These are the knowledge gaps that motivated this study—we sought to understand the geographic and environmental factors that determine the amounts and types of NTM found in showerhead biofilms. Likewise, given the potential for these bacteria to cause respiratory disease, we investigated whether there is a concordance of the geographic distributions of showerhead mycobacteria with the prevalence of NTM lung disease, the latter estimated from two separate epidemiological surveys.

## RESULTS AND DISCUSSION

### Mycobacterial abundance across showerheads.

We worked with citizen scientists to collect showerhead biofilm samples from locations across the United States and Europe, with 656 samples included in downstream analyses (606 from the United States and 50 from Europe; see Fig. S1 in the supplemental material). We extracted DNA directly from these biofilm samples and used a cultivation-independent 16S rRNA gene sequencing approach to assess overall bacterial community composition in each sample. We found that bacteria assigned to the genus *Mycobacterium* represented, on average, the most abundant group of bacteria found in the showerhead biofilm samples (Fig. S2). Other abundant taxa included members of the genera *Sphingomonas*, *Bradyrhizobium*, *Blastomonas*, and *Phenylobacterium*, bacterial taxa commonly observed in water distribution systems (1, 15, 31). Across all samples, the mean abundance of the *Mycobacterium* genus was 13.5% of 16S rRNA gene reads (Fig. S2). However, the abundances of mycobacteria were highly variable across the samples, ranging from 0% (no mycobacteria detected) to >99% of bacterial 16S rRNA gene reads, with *Mycobacterium* accounting for >10% of 16S rRNA gene reads in 238 of the total 652 samples. The observed ubiquity of mycobacteria is comparable to that observed in other studies that have used cultivation-independent methods to assess mycobacterial abundances in water distribution systems (4, 14, 15, 31).

10.1128/mBio.01614-18.1 FIG S1 Map of the households from which showerhead biofilm samples were collected for this study (*n *=* *656). The yellow points indicate households on municipal water (*n *=* *520), while the green points indicate households on well water (*n *=* *86). Download FIG S1, PDF file, 0.2 MB. Copyright © 2018 Gebert et al. 2018 Gebert et al. This content is distributed under the terms of the Creative Commons Attribution 4.0 International license.

10.1128/mBio.01614-18.2 FIG S2 Box and whisker plots showing the dominant bacterial genera detected in 652 showerheads. Bars indicate mean abundances of each genus across all samples; the color of each bar indicates the broader taxonomic affiliation of each genus. Download FIG S2, PDF file, 1.3 MB. Copyright © 2018 Gebert et al. 2018 Gebert et al. This content is distributed under the terms of the Creative Commons Attribution 4.0 International license.

**FIG 1:**
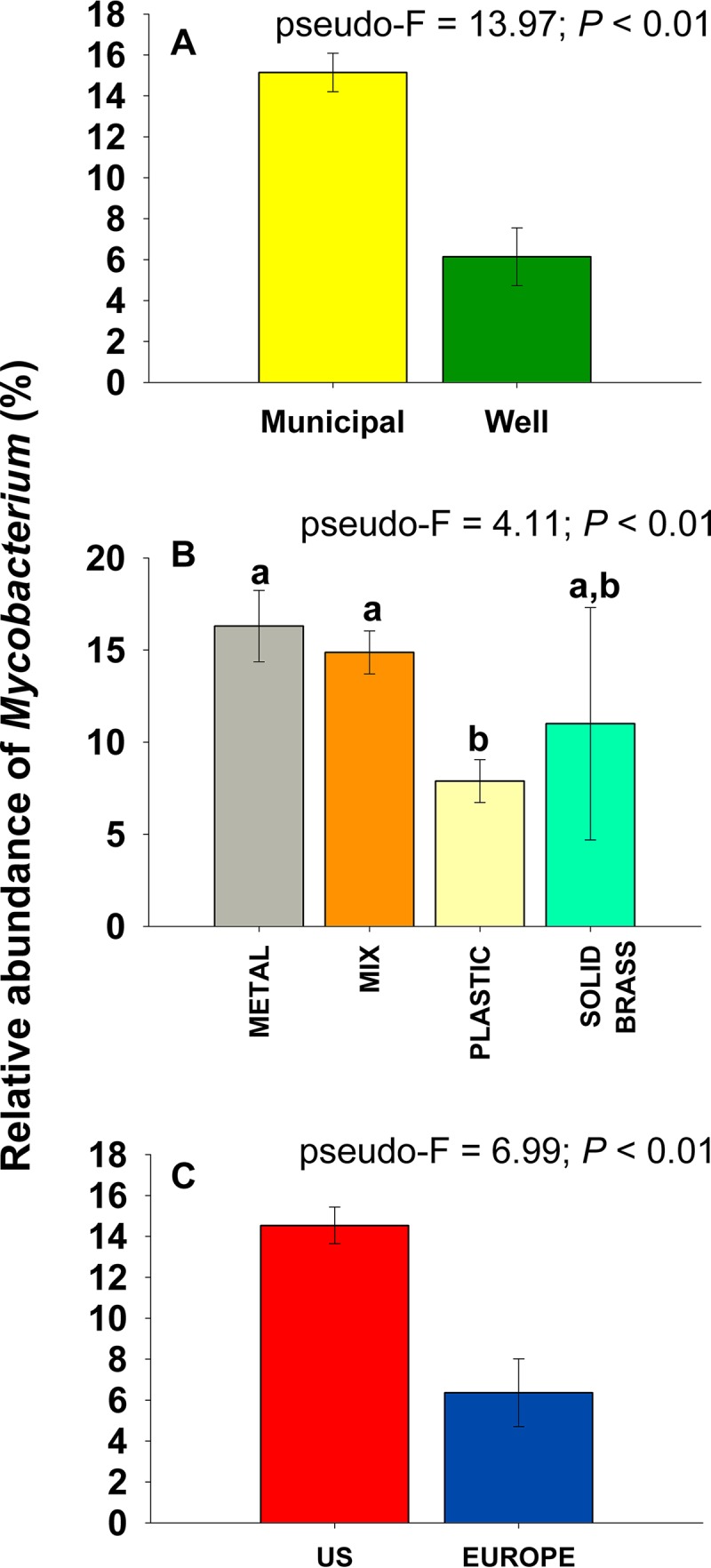
Differences in the relative abundances of mycobacteria (as determined via 16S rRNA gene sequencing) across households in the United States on municipal versus well water (A), across showerheads constructed of different materials (B), and across households in the United States versus Europe (C).

Overall, mycobacteria were more than two times more abundant in U.S. homes receiving water from municipal water treatment plants than in homes on well water (*P < *0.001; Fig. 1A). Water treatment plants in the United States must maintain excess chlorine-based disinfectant within the distribution system (32), and we found, as expected, that those homes on municipal water had measured total chlorine concentrations 15 times higher, on average, than homes with well water (Fig. S3). The observation of higher abundances of mycobacteria in showerheads receiving municipal water than in those receiving well water is consistent with previous work (15) and was likely a result of disinfection selecting for mycobacteria, as they are typically more resistant than other bacteria to the toxic effects of chlorine and chloramine (24, 31, 33). We also found that the relative abundances of mycobacteria in plastic showerheads were, on average, two times lower than in showerheads that were constructed of either metal or a mix of metal and plastic components (*P < *0.01; Fig. 1B). Similar results have been reported previously (34, 35), and these patterns are likely a product of the leaching of biodegradable carbon from plastic materials supporting elevated growth of other bacterial taxa that can outcompete mycobacteria in showerhead biofilms (1, 36).

10.1128/mBio.01614-18.3 FIG S3 Differences in shower water chemistry across the U.S. households on either municipal or well water, as measured by the participants in this study. Download FIG S3, PDF file, 1.1 MB. Copyright © 2018 Gebert et al. 2018 Gebert et al. This content is distributed under the terms of the Creative Commons Attribution 4.0 International license.

It has been suggested that mycobacteria should be less abundant in biofilm samples dominated by methylotrophs, particularly *Methylobacterium* spp. (37, 38). In contrast to this expectation, we found that showerheads with higher abundances of *Methylobacterium* did not necessarily have lower proportional abundances of mycobacteria (Fig. S4). Therefore, *Methylobacterium* is unlikely to be a useful indicator of mycobacterial loads. We did, however, identify a positive correlation between mycobacterial abundances and the abundances of free-living amoebae (FLA) within the Vermamoebidae group in a subset of samples (*n *=* *89) for which we obtained both bacterial and eukaryotic small-subunit rRNA gene sequence data (Pearson’s *r* = 0.25, *P = *0.02). This pattern was mainly driven by *Vermamoeba* (*Hartmannella*) *vermiformis*; it was the most abundant microeukaryote species detected in our samples and is commonly found in water distribution systems (30, 39) and was previously shown to be capable of harboring intracellular mycobacteria (28, 29). The apparent co-occurrence of FLA and mycobacteria highlights the importance of considering potential associations between protists and bacteria in trying to predict distributions of mycobacteria and other potentially pathogenic bacteria (e.g., *Legionella*) in residential water systems (40–42).

10.1128/mBio.01614-18.4 FIG S4 The relative abundances of *Methylobacterium* versus *Mycobacterium* across all samples for which we obtained 16S rRNA gene sequence data (*n *=* *652). Download FIG S4, PDF file, 0.3 MB. Copyright © 2018 Gebert et al. 2018 Gebert et al. This content is distributed under the terms of the Creative Commons Attribution 4.0 International license.

The genus *Mycobacterium* was not equally abundant across all geographic regions included in our sampling effort (Fig. S5). Strikingly, the abundance of taxa assigned to the genus *Mycobacterium* in showerhead biofilms in the United States was, on average, 2.3 times higher than that measured for showerheads in Europe (*P* <0.01; Fig. 1C). The lower abundances of mycobacteria in Europe than in the United States may be driven by a myriad of interacting factors, including differences in water treatment, water heating systems, source water type, showerhead design, and characteristics of the water distribution systems (23). However, our analyses of shower water chemistry by the citizen scientists indicate that the observed differences may be driven, in part, by differences in water chemistry, as we found that U.S. households receiving municipal water had significantly higher chlorine and iron concentrations, but significantly lower pH and nitrate levels, than European households on municipal water (Fig. S6). Most notably, total chlorine concentrations in shower water in the United States were 11 times higher, on average, than those measured in shower water in the European households (Fig. S6). The practice of adding chlorine-based residual disinfectants during water treatment is less common in Europe than in the United States (43), and this key difference in water treatment practices may be one of the factors contributing to the elevated abundances of mycobacteria observed in showerheads from U.S. households.

10.1128/mBio.01614-18.5 FIG S5 Relative abundances of the genus *Mycobacteria* across the 21 geographic clusters (regions) that had at least 10 samples per region. The differently colored points on the map correspond to each of the 21 regions, and the unfilled points indicate households that did not fall into any of these 21 geographic clusters. Download FIG S5, PDF file, 0.4 MB. Copyright © 2018 Gebert et al. 2018 Gebert et al. This content is distributed under the terms of the Creative Commons Attribution 4.0 International license.

10.1128/mBio.01614-18.6 FIG S6 Differences in shower water chemistry across the U.S. versus European households. Only those households on municipal water supplies were included in these analyses (*n *=* *568 in total [520 from the United States and 48 from Europe]). Download FIG S6, PDF file, 1.0 MB. Copyright © 2018 Gebert et al. 2018 Gebert et al. This content is distributed under the terms of the Creative Commons Attribution 4.0 International license.

### Mycobacterial diversity in showerheads.

**FIG 2:**
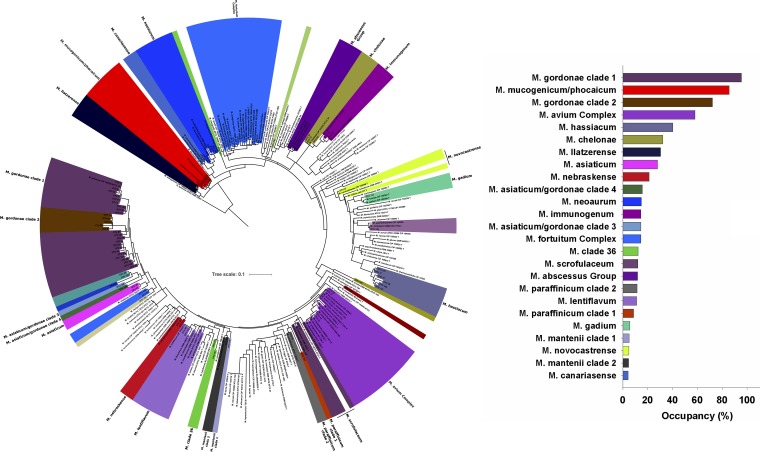
Phylogenetic tree showing the mycobacterial diversity recovered from the cultivation-independent analyses (hsp65 gene sequencing). Included in the tree are the reference mycobacterial strains from Dai et al. (59). The colors indicate the 34 clades of *Mycobacteria*, with the labels indicating the taxonomic identity of each clade. The tree was rooted with a hsp65 sequence from Nocardia farcinica (DSM43665). The plot on the right shows percent occupancy of the top 25 mycobacterial clades, with occupancy assessed as the percentage of samples (among 656 in total) in which each clade was detected. Colors indicate unique mycobacterial clades, with the color scheme used in the tree matching the color scheme used in the associated plot of mycobacterial occupancy.

**FIG 3:**
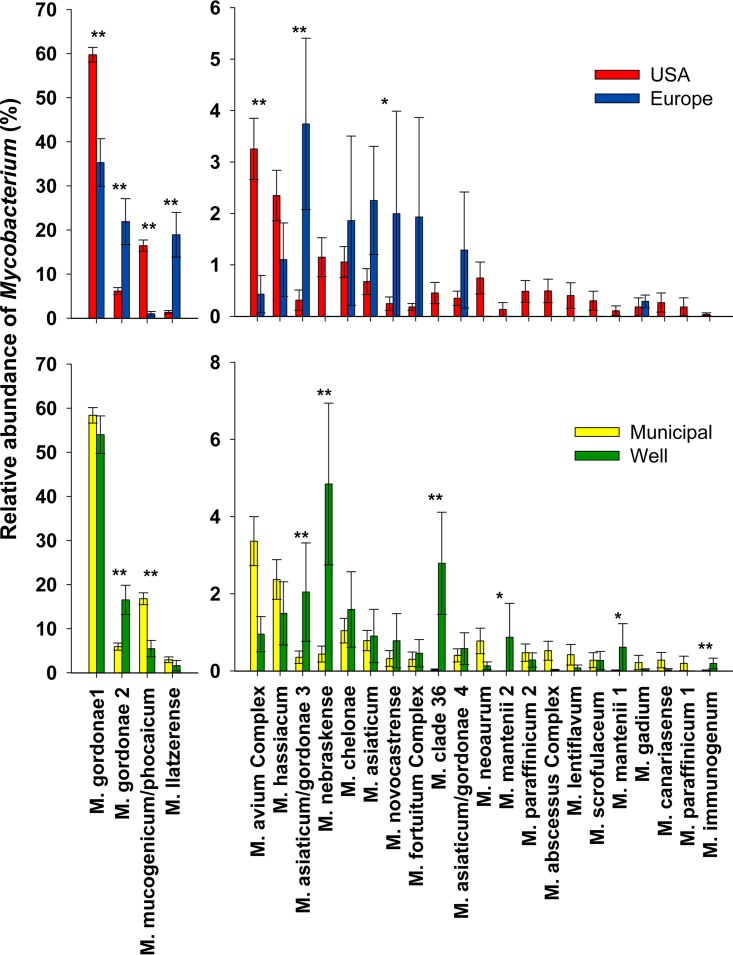
Abundances of the top 25 mycobacterial clades detected across homes in the United States on well versus municipal water (*n *=* *520 and 86, respectively; top panel) and across homes in the United States versus Europe (municipal water only, *n *=* *606 and 50, respectively; bottom panel). The *y* axes were split to better illustrate the differences among the less-abundant clades.

The aforementioned analyses focused on measured abundances of the genus *Mycobacterium*, as inferred from 16S rRNA gene sequence data. However, the genus *Mycobacterium* includes nearly 200 species that can differ with respect to their ecologies and pathogenicity (44). Thus, to obtain more-detailed information on what specific mycobacterial species, including potential pathogens, were found in the showerhead biofilms, we amplified DNA from each of the 656 showerhead samples and sequenced a portion of the hsp65 (65-kDa heat shock protein) gene using mycobacterium-specific primers (see Materials and Methods). In total, we recovered 1,029 mycobacterial exact sequence variants (ESVs), with the 100 ESVs presented in Fig. 2 accounting for >95% of the reads. These ESVs span nearly the full extent of known mycobacterial diversity, highlighting that biofilms of household water distribution systems can harbor an extensive array of mycobacterial species, including a number of taxa that are not closely related to any previously described isolates (Fig. 2). We combined the top 100 ESVs into 34 phylogenetically defined clades for downstream analyses. We found that the dominant mycobacterial clades recovered from the showerhead biofilms included potential pathogens that are frequently recovered from patients diagnosed with NTM infections (e.g., M. avium complex, M. abscessus complex, M. fortuitum complex) as well as mycobacteria that are not typically considered pathogenic (e.g., M. gordonae, M. hassiacum, M. canariasense) (Fig. 2). Information on the ubiquity and median abundance of the dominant mycobacterial clades detected in the showerheads using our cultivation-independent hsp65 sequencing approach is provided in Fig. 2 and 3, respectively.

Most of the mycobacterial diversity found in the showerhead biofilm samples was not captured in a corresponding cultivation-dependent survey. Among those 186 samples which were analyzed using both cultivation-dependent and cultivation-independent approaches, we identified nearly four times more mycobacterial ESVs with the cultivation-independent approach (Fig. S7). Many clades (including some of the more abundant clades, such as the M. gordonae, M. hassiacum, and M. llatzerense clades) were missed completely with the cultivation-dependent approach (Fig. S7). While some mycobacterial clades were detected with both approaches (Fig. S7), culturing captured only a small fraction of the total mycobacterial diversity found in showerheads. These findings confirm results from previous studies (26, 45) that indicated that many mycobacteria in the environment (and possibly in patients with respiratory infections) are simply missed by the use of standard culture techniques. Our results highlight the importance of using cultivation-independent approaches for detecting mycobacteria when possible, as the mycobacterial diversity in these biofilm samples, and other sample types, is likely to be significantly underestimated in studies relying on cultivation-based surveys.

10.1128/mBio.01614-18.7 FIG S7 Phylogenetic tree indicating those hsp65 exact sequence variants (ESVs) detected in 186 samples for which we had conducted both cultivation-independent and cultivation-dependent assessments of mycobacterial diversity. Individual ESVs are colored by detection in both cultivation-independent and cultivation-dependent assays (orange), detection using the culture-independent approach only (blue), and detection in culture only (pink). No color indicates either a database (reference) sequence or an ESV that was detected in another sample (not one of the 186 samples used for the culture-independent versus culture-dependent comparison). The bar plot at the bottom indicates the relative abundances of the dominant clades detected in the cultivation-independent analyses of DNA extracted directly from the environmental samples (showerhead biofilms—black bars) versus analyses of the DNA extracted from the cultivated mycobacterial isolates (gray bars). Download FIG S7, PDF file, 0.8 MB. Copyright © 2018 Gebert et al. 2018 Gebert et al. This content is distributed under the terms of the Creative Commons Attribution 4.0 International license.

### Biogeography of selected mycobacteria.

**FIG 4:**
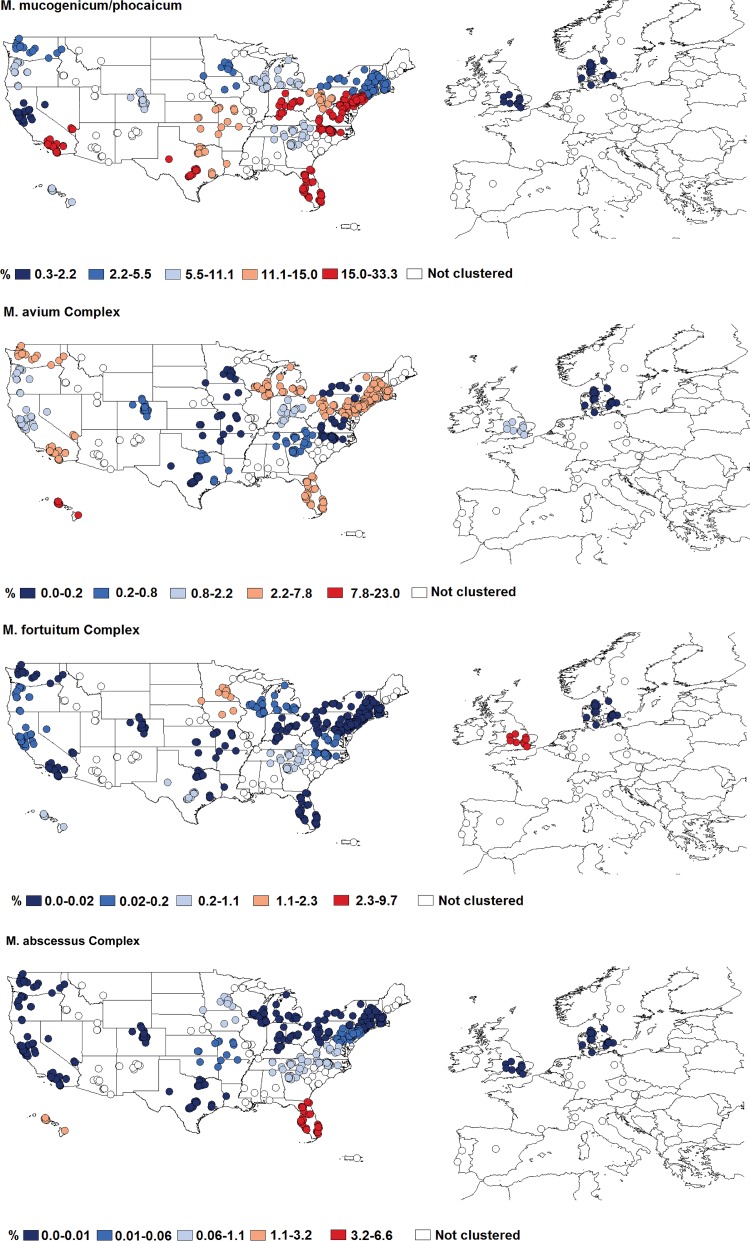
Differential abundances of each of four lineages of mycobacteria that include pathogens across the geographic clusters of showerhead biofilm samples. The different colors indicate the mean abundances of each mycobacterial lineage across each of the 23 geographic clusters identified (uncolored points are samples from households not included in any of these 23 clusters). For details on each cluster and the abundances of mycobacteria within each cluster, see Fig. S8.

The individual mycobacterial clades detected by our cultivation-independent hsp65 gene sequencing analyses, including clades with known pathogens, often exhibited distinct geographic patterns. Not all mycobacterial lineages were likely to be found everywhere—the mycobacterial diversity found in showerheads varied as a function of household location (Fig. 3 and 4; see also Fig. S8 and S9). Some clades were far more abundant in Europe than in the United States, including multiple clades related to M. gordonae and a clade that included M. llatzerense, which was 14 times more abundant in Europe than in the United States (Fig. 3). We note that a study of mycobacteria in Parisian tap water systems also found M. llatzerense to be dominant, even though this species is rarely detected in the United States (23, 46). In contrast, we found mycobacteria within the M. avium complex (MAC), which includes multiple opportunistic pathogens, were relatively more abundant in U.S. showerheads than in those from Europe. Although these differences in the abundances of specific mycobacterial groups between U.S. and European households may be a product of dispersal limitation, we expect that these patterns are more likely driven by differences in water chemistry (Fig. S6), water distribution systems, or water treatment practices. Although showerheads are only one potential source of NTM infections, the significant differences in the mycobacterial communities inhabiting U.S. versus European showerheads may explain some of the documented geographic variation in the clinical isolates obtained from NTM patients on the two continents (22).

10.1128/mBio.01614-18.8 FIG S8 Relative abundances of mycobacterial clades across the 21 geographic clusters (regions) that had at least 10 samples per region. The differently colored points on the map correspond to each of the 21 regions, and the unfilled points indicate households that did not fall into any of these 21 geographic clusters. Only those clades that exhibited significant differences in relative abundance across regions (*P < *0.05) are shown here. For information on the geographic distributions of the remaining abundant clades, see Fig. S9 Download FIG S8, PDF file, 2.8 MB. Copyright © 2018 Gebert et al. 2018 Gebert et al. This content is distributed under the terms of the Creative Commons Attribution 4.0 International license.

10.1128/mBio.01614-18.9 FIG S9 Relative abundances of the remaining mycobacterial clades (those not shown in Fig. S8) across the 21 geographic clusters (regions) that had at least 10 samples per region. The differently colored points on the map correspond to each of the 21 regions, and the unfilled points indicate households that did not fall into any of these 21 geographic clusters. For the 17 clades shown here, none exhibited statistically significant (*P < *0.05) variations in abundances across the 21 geographic clusters. Download FIG S9, PDF file, 0.7 MB. Copyright © 2018 Gebert et al. 2018 Gebert et al. This content is distributed under the terms of the Creative Commons Attribution 4.0 International license.

Within the United States, households supplied by municipal versus well water had distinct mycobacterial communities (Fig. 3). Most notably, homes on municipal water had higher abundances of the M. mucogenicum/M. phocaicum clade, potentially pathogenic mycobacteria (47). Other mycobacterial taxa, including M. nebraskense and some M. gordonae clades, were more abundant in U.S. homes that use well water. However, these taxa are rarely considered pathogenic (10, 12, 48), suggesting that levels of exposure to pathogenic mycobacteria from showerheads are typically higher in homes on municipal water. This finding is consistent with reports that water source has been found to play a role in NTM disease prevalence (24, 49).

Some of the mycobacterial lineages that include known pathogens were more likely to be abundant in showerheads from certain regions of the United States (Fig. 4; see also Fig. S8 and S9). We found that four mycobacterial clades that are often considered pathogenic (M. mucogenicum*/*M. phocaicum, the M. avium complex, the M. fortuitum complex, and the M. abscessus complex) exhibited significant geographic variation in their abundances across the United States (Fig. 4). While these four pathogenic clades exhibited distinct geographic patterns, showerheads from homes in Hawaii, southern California, Florida, the upper Midwest, and the mid-Atlantic states consistently had higher abundances of one or more of these pathogenic mycobacteria. Most notably, bacteria of the M. abscessus complex and M. avium complex were far more abundant in Hawaii, Florida, and the northeastern United States than in other regions. The geographic hot spots in mycobacterial abundances shown in Fig. 4 were, to some degree, predictable from shower water chemistry. The abundances of these pathogenic mycobacterial lineages in showerheads were often significantly correlated with mean annual outdoor temperature and shower water total chlorine, alkalinity, and pH levels, with the specific direction of these correlations depending on the clade in question (Fig. S10). Most notably, showerheads found in households from warmer locations with higher shower water chlorine concentration levels were found to have higher abundances of the M. mucogenicum*/*M. phocaicum clade. However, much of the observed geographic variation in the abundances of other mycobacterial groups (including the M. abscessus complex and the M. avium complex) could not be reconciled from the variables included in our model (Fig. S10). This may be because we did not measure all of the relevant shower water characteristics (e.g., organic carbon concentrations, water heater temperature) or because the conditions that favor the growth of these mycobacteria are determined by source water characteristics or conditions at other points in the water distribution network. Nevertheless, we did find that individual mycobacterial lineages have distinct geographic distributions and environmental preferences, information that can be used to improve predictions of which showerheads are most likely to harbor pathogenic mycobacteria.

10.1128/mBio.01614-18.10 FIG S10 Semipartial correlations (Spearman) indicating the correlations between water chemistry parameters and mean annual temperature (MAT) with the relative abundances of mycobacteria (16S rRNA gene sequence data) and individual mycobacterial clades (hsp65 sequence data). These analyses were conducted using all samples (both U.S. and European samples) included in this study. Download FIG S10, PDF file, 2.7 MB. Copyright © 2018 Gebert et al. 2018 Gebert et al. This content is distributed under the terms of the Creative Commons Attribution 4.0 International license.

### Showerhead-associated mycobacteria and the epidemiology of NTM lung disease.

**FIG 5:**
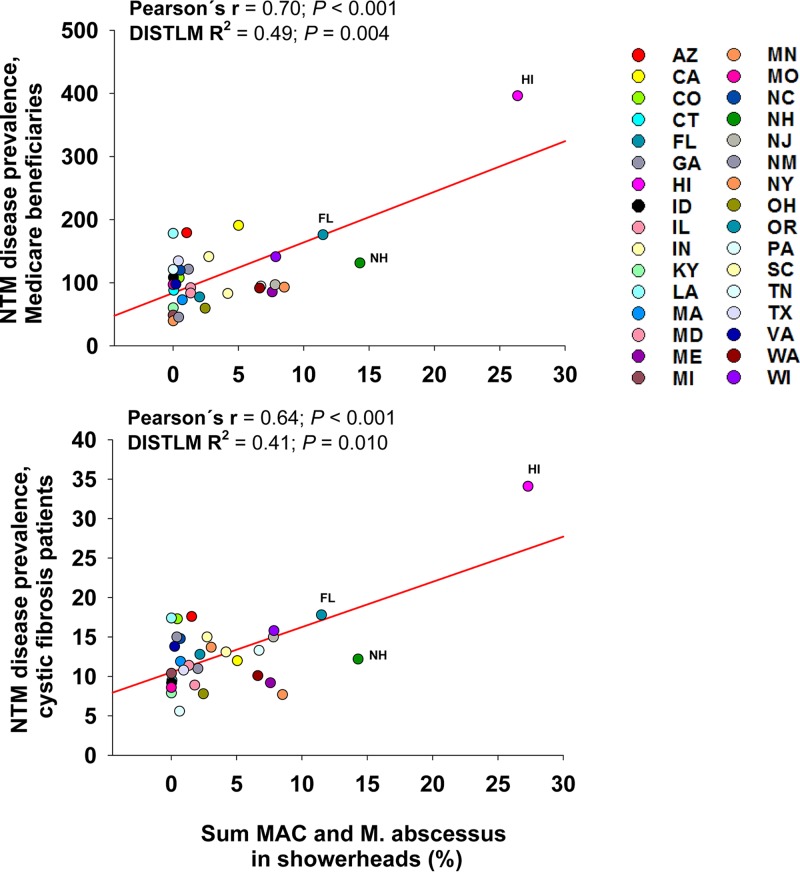
Relationships between the summed relative abundances of three potentially pathogenic mycobacterial clades that were frequently detected in showerheads (M. abscessus, M. fortuitum, and MAC) and the reported prevalences of NTM disease across Medicare beneficiaries and cystic fibrosis patients (50, 70). The strength and significance of the correlations were tested using both Pearson correlations and distance-based linear models (DISTLM; output shown). Each point indicates a different U.S. state, and data were aggregated to the state level (using median abundances), as the disease prevalence data were available only at the state level of resolution. Only states with >10 showerhead samples were included in these analyses.

Those regions within the United States that we identified as having relatively high abundances of known mycobacterial pathogens in showerhead biofilms (Fig. 4) generally overlapped the regions in the United States previously reported to have a higher-than-average prevalence of NTM lung disease (20). For example, some of the U.S. regions with the highest reported incidences of patients diagnosed with NTM lung disease (including Florida, Hawaii, southern California, and the mid-Atlantic states) are also the regions where we found showerheads with high abundances of potentially pathogenic mycobacteria (Fig. 4). To further investigate this pattern, we explicitly tested whether the distributions of pathogenic NTM across the U.S. showerheads in this study were correlated with geographic patterns in NTM lung disease prevalence. We compared the state-level median abundances of two potentially pathogenic mycobacterial clades that were frequently detected in showerheads (M. abscessus complex and M. avium complex) to the reported prevalences of NTM lung disease across Medicare beneficiaries and persons with cystic fibrosis (Fig. 5). We expected only a modest correlation, if any, given the relatively small number of showerheads per state and the constraints associated with accurately estimating NTM lung disease prevalence (20, 50). However, we found significant correlations between the abundances of these mycobacteria found in showerheads sampled across the United States and NTM lung disease prevalence, the latter data having been derived from two independent data sets (Pearson’s *r* = >0.6 and *P* = *<*0.001 in both cases) (Fig. 5). These results add to the growing body of evidence suggesting that mycobacteria living in showerheads are likely an important source of NTM infections.

### Conclusions.

Mycobacteria are frequently abundant in showerheads, and many showerheads harbor mycobacterial lineages that include known pathogens. The amounts and types of mycobacteria found in showerheads appear to vary depending on household location, water source, water chemistry, the presence or absence of free-living amoebae, and showerhead material. Moreover, those geographic regions with higher abundances of mycobacterial pathogens tend to be hot spots of NTM lung disease within the United States. So far, our results are correlative, but they are in line with previously published work on the factors structuring mycobacterial abundances and the importance of showerheads as a source of NTM infections. We also demonstrate the synergy of coupling an extensive citizen scientist sampling effort with molecular diagnostic techniques to comprehensively investigate the factors associated with the likelihood of acquiring NTM lung disease. More generally, our results highlight the relevance of understanding how shifts in household water sources (i.e., from well to municipal water sources) and water treatment practices may be contributing to the apparent rise in NTM infections in U.S. and European populations.

## MATERIALS AND METHODS

### Sample collection.

Showerhead biofilm samples were collected by citizen scientists participating in the Showerhead Microbiome Project (goo.gl/7G6xbc). We recruited participants using the website, social media, and email campaigns from throughout the United States and Europe from July 2016 to November 2016. Enrolled participants were provided a written Informed Consent form approved by the Human Research Committee of North Carolina State University (approval no. 9158). Each participant was then provided with a sampling kit that contained a dual-tipped sterile Puritan CultureSwab, a water chemistry analysis kit, sterile gloves to be worn during sampling, and a brief questionnaire. Participants were not queried about their NTM infection status. All biofilm samples were collected by swabbing the interior of an unscrewed showerhead, with participants asked to swab the most commonly used showerhead in each household as close to the inside interface of the showerhead as possible. Each participant was asked to provide the household address, the household water source, the estimated time since installation of showerhead, its usage frequency, cleaning frequency, and a description of the showerhead sampled (including materials and spray pattern). All swab samples collected from the United States were mailed directly to the University of Colorado, where they were stored in a −20°C freezer until processing. Swab samples from Europe were mailed directly to Copenhagen, Denmark, where they were stored at −20°C until the European collection was completed. The European swab samples were then sent overnight on dry ice to the University of Colorado, where they were stored at −20°C until processing. In total, we collected 691 samples, including 638 showerhead biofilm samples from across the United States (49 of 50 U.S. states) and 53 samples from Europe (13 different countries) (see Fig. S1 in the supplemental material).

### Water chemistry analyses.

Each participant conducted basic chemical analyses of the water collected from the same shower used for the biofilm sample collection. Water chemistry was determined for each showerhead using Hach Aquachek water quality test strips (Hach, Loveland, CO, USA). Each kit included a “5-in-1” test strip (which measures total chlorine, free chlorine, total hardness, total alkalinity, and pH), a test strip for nitrate and nitrite, and a test strip for total iron concentrations.

### 16S rRNA gene sequencing to characterize showerhead bacterial communities.

We used an approach described previously (51) to amplify and sequence the V4 hypervariable region of the 16S rRNA gene from all 691 biofilm samples. DNA was extracted from one of the two swabs collected per showerhead using a Qiagen PowerSoil DNA extraction kit and was then PCR amplified in duplicate reactions using the 515f/806r primer pair modified to include Illumina adapters and the appropriate error-correcting barcodes (52). Each 25-µl reaction mixture included 12.5 µl of Promega HotStart Mastermix, 10.5 µl of PCR-grade water, 1 µl of PCR primers (combined at 10 µM), and 1 µl of purified genomic DNA.

The thermocycler program consisted of an initial step at 94°C for 3 min followed by 35 cycles of 94°C for 45 s, 50°C for 1 min, and 72°C for 1.5 min. The program concluded with a final elongation step at 72°C for 10 min. Both “no-template” controls and “DNA extraction kit” controls were included with each set of 90 samples to check for potential contamination. Duplicate reactions were pooled, cleaned, and normalized using a Thermo Fisher SequalPrep normalization plate kit. Amplicons were sequenced on 3 MiSeq runs at the University of Colorado Next-Generation Sequencing Facility with 2-by-150-bp paired-end chemistry.

After demultiplexing and combining the data from all 3 sequencing runs, paired reads were merged with a minimum length of 200 bp for the merged sequence and quality filtered using the uSearch10 pipeline (53), discarding sequences with greater than 1 error per base call. The quality-filtered reads were processed using uNoise3 (54) to identify exact 16S rRNA gene sequence variants (ESVs). Taxonomy was determined for each ESV using the Ribosomal Database Project Classifier (55) trained on the Greengenes database (56). After removing those ESVs that were represented by <25 reads across the entire data set and those classified as mitochondria or chloroplasts, we ignored any samples that yielded fewer than 2,000 reads per sample. A total of 39 of the 691 biofilm samples and all of the no-template and DNA extraction kit negative controls failed to meet this threshold for sequencing depth and were excluded from downstream analyses. The percentage of mycobacteria in each sample was calculated by summing all reads classified in the *Mycobacterium* genus.

### *Mycobacterium*-specific hsp65 sequencing.

As the 16S rRNA gene sequencing analyses described above provide insufficient resolution to differentiate among many mycobacterial lineages, we also analyzed all biofilm samples by PCR amplifying and sequencing a region of the hsp65 gene using mycobacterium-specific PCR primers (57) and included negative controls in all analyses as described above.

A “two-step PCR” protocol was used for the hsp65 amplifications with sample-specific barcodes ligated to the hsp65 amplicons in a second round of PCR. The first PCR step was conducted with duplicate reactions per extracted DNA sample using the Tb11/Tb12 primers (57) that included the appropriate Illumina adapters. Each 25-µl PCR recipe was identical to that described above with the following thermocycler program: a 3-min initial step at 94°C followed by 45 cycles of 94°C for 60 s, 60°C for 60 s, and 72°C for 60 s, with a final 10-min elongation step. Duplicate reaction mixtures were pooled and the amplicons were cleaned using a Qiagen UltraClean PCR cleanup kit following the instructions of the manufacturer.

A second round of PCR was then performed to attach a unique 12-bp error-correcting barcode to the amplicons from each sample to allow multiplexing. Each of the 42-µl reaction mixtures included 20 µl of Promega Hotstart Mastermix and 14 µl of PCR-grade H_2_O, with 4 µl of the forward/reverse universal 12-bp barcodes (10 µM each) and 2 µl of the Tb11-Tb12 amplicons from the previous round of PCRs added to 36 μl of the Mastermix and H_2_O mixture. The thermocycler program included an initial step at 95°C for 3 min followed by 8 cycles of 95°C for 30 s, 55°C for 30 s, and 72°C for 30 s, with a final step at 72°C for 5 min. The resulting barcoded hsp65 amplicons were then cleaned and normalized using the method described above. Amplicons were sequenced in 2 Illumina MiSeq runs using the 2-by-300-bp paired-end chemistry at the University of Colorado Next-Generation Sequencing Facility.

After the reverse reads were demultiplexed and trimmed to 250 bp using fastq_filter, the paired reads were merged with a minimum length of 200 bp for the merged sequence and quality filtered using the uSEARCH10 pipeline (53), discarding those sequences with >1 error per base call. We used the uNOISE3 pipeline (58) to identify exact sequence variants (hsp65 ESVs) with the taxonomy assigned using the Ribosomal Database Project Classifier (55) trained on the hsp65 reference database described by Dai et al. (59). The representative sequences for each ESV were then compared to the 157 hsp65 sequences in the reference database compiled by Dai et al. (59) using the BLAST algorithm (60). Those ESVs that did not have >90% similarity to sequences in the reference database were removed as they were not from members of the *Mycobacterium* genus. Across the whole data set, this process removed 5% of the quality-filtered hsp65 reads, with most of the removed reads classified as belonging to other genera within the *Actinomycetales* order. Only those samples with >200 quality-filtered mycobacterial hsp65 reads per sample were included in downstream analyses, and this threshold removed 5 of the 691 biofilm samples and all of the no-template and DNA extraction kit negative controls.

We focused our downstream analyses on the top 100 mycobacterial ESVs across the whole mycobacterium filtered data set (these ESVs accounted for 96% of the quality-filtered mycobacterial hsp65 reads). The 100 representative hsp65 ESV sequences were aligned against the hsp65 reference available in the Dai et al. (59) database using MUSCLE v.3.8.31 (61), and a phylogenetic tree was constructed using RAxML (62) with a hsp65 sequence from Nocardia farcinica (DSM43665) to root the tree. All of the ESVs in the best-scoring phylogenetic tree were clustered into discrete clades using RAMI (63) with the patristic distance threshold set to 0.05 (clades defined as having >5% patristic distance across the sequenced portion of the hsp65 gene). The taxonomic identity of each clade was determined based on sequence similarity to the type strains that fell within each clade and the phylogenetic tree as visualized using iTOL (64). Information on each of the 100 hsp65 ESVs, their assigned clades and phylogenetic placement, and their similarity to type strains is included in Fig. 3.

### Protistan analyses.

As mycobacteria are known to associate with various protists, we wanted to test for patterns of co-occurrence between mycobacteria and specific protists. To do so, we selected a subset of 186 samples for which we obtained 18S rRNA marker gene data following protocols as described previously by Ramirez el al. (65). In brief, we used the 1391f/EukBr primer set and pooled and sequenced amplicons along with appropriate controls as described above for 16S rRNA communities. Reads were demultiplexed, merged, and trimmed to 100-bp lengths and quality filtered as described above. A database of ≥97% similar sequence clusters was constructed using USEARCH (53) and taxonomic assignments were made with the PR2 database (66). Of the 186 samples, we retained 89 samples for downstream analyses with a minimum threshold of 1,200 reads per sample, after removing nonmicrobial eukaryotes, including Streptophyta and Metazoa. To account for differential read coverage, we rarefied samples to 1,200 reads per sample for the 18S rRNA analyses and also rarefied the 16S samples to 1,500 reads per sample for the subset of 89 samples for which we obtained protist data.

For the subset of samples from which we obtained both 16S and 18S rRNA gene data (*n* = 89), we investigated whether the relative abundances of the most dominant groups of free living amoebae (FLA) were correlated with mycobacterial relative abundances (at the genus level). To do this, we conducted Pearson’s correlations to evaluate the correlations among the three most abundant FLA families (Vermamoebidae, Acanthamoebidae, and Echinamoebidae) with the relative abundances of the genus *Mycobacterium* across the 89 samples.

### Cultivation-based analyses of mycobacteria.

In addition to the cultivation-independent DNA sequencing-based analyses described above, 186 randomly selected biofilm samples were cultured using a standard approach for the culture and isolation of environmental mycobacteria (36). Briefly, swabs were immersed in 2 ml of autoclaved ultrapure water and subjected to vortex mixing on the high setting for 1 min. To select for mycobacteria, 450 µl of sample was transferred to a sterile Eppendorf tube with 50 µl of 1% cetylpyridinium chloride, subjected to vortex mixing, and incubated at room temperature for 30 min. After incubation, 100 µl of each sample was plated on duplicate Middlebrook 7H10 agar with oleic acid/glycerol enrichment and incubated at 37°C for 21 days. Among the 186 samples cultured, 40 were positive for mycobacteria (no mycobacterial isolates were recovered from the remaining 146 samples). Thus, we were able to obtain mycobacterial isolates from 40 samples, with these 40 samples yielding 74 unique isolates. We extracted DNA from all 74 isolates, in addition to a “blank” control sample of the media used to grow the isolates, using a Qiagen Powersoil DNA extraction kit, and sequenced the amplified hsp65 to identify each isolate using the approach described above. All isolates had >98% sequence similarity matches over the entire length of the amplicon to the sequences in the Dai et al. (59) database. The hsp65 sequences from these isolates were placed into the phylogenetic tree described above, including the reference sequences and the sequences obtained from the cultivation-independent analyses of all the biofilm samples. We then assigned the isolate sequences to their respective lineages and determined their taxonomic identity using the methods described above. Information on each of these isolates, their taxonomic identities, and their phylogenetic placement is provided in Fig. S7.

### “Storage” study.

We conducted a separate experiment to determine how prolonged storage at room temperature affects the showerhead biofilm bacterial communities. We did this to determine whether shipping samples unrefrigerated from across the United States to Boulder, CO, might have influenced our determination of mycobacterial relative abundances. For this experiment, we collected 12 replicate swabs placed in individual showerheads at each of 7 households (2 in Colorado, 2 in Hawaii, 2 in North Carolina, and 1 in southern California). These swabs were cut 2.5 cm from the tip and placed into the accessible faceplate of 7 “polished brass” showerheads provided by Shower Clear (West Orange, NJ, USA). Participants were asked to replace the preexisting showerheads in each of the 7 households for 30 to 40 days. After this time period, the replicate swabs from each showerhead were shipped to the University of Colorado overnight at 4°C, where they were either frozen at −20°C immediately (day 0) or held under room temperature conditions inside sealed bags (to minimize drying) for 3, 7, and 10 days prior to freezing at −20°C. Genomic DNA was extracted from all swabs (7 homes, 12 replicate swabs per home, 3 per storage duration), and the V4 region of the 16S rRNA gene was amplified and sequenced from the extracted DNA using the protocol described above. We used the pipeline described above to calculate the relative abundances of *Mycobacterium* (percent quality-filtered 16S rRNA gene sequences) on each of the swabs, after rarefying all samples to 8,000 reads per sample. Twenty samples from North Carolina and all of the negative-control samples (both extraction blanks and no-template controls) were discarded due to insufficient sequencing depth (<1,000 reads per sample), yielding a total of 64 swab samples included in downstream analyses. We used a permutational multivariate analysis of variance (PERMANOVA) test (as implemented in the R package vegan) to determine if storage duration had a significant effect on the estimated mycobacterial abundances, with sampling location (household) set as a fixed variable and unfrozen storage time as a random variable. We found no significant influence of storage time on the relative abundances of *Mycobacterium* in these samples (pseudo-F = 1.67, *P > *0.20). Thus, we can conclude that potential differences in the duration of time samples spent unfrozen in transit are unlikely to have influenced our estimates of mycobacterial abundances on swabs collected as part of the broader sampling effort.

### Statistical analyses and predictive modeling.

Of the 691 samples collected from across the United States and Europe, only 656 were included in downstream analyses. Thirty-five samples were excluded because corresponding information on household location (latitude/longitude), water source (municipal versus well water), frequency of showerhead usage, or showerhead material was not provided. Likewise, 37 of the 656 samples were excluded from the analyses of mycobacterial abundances (genus level) because insufficient 16S rRNA gene sequence data were available for those samples. For the water chemistry analyses, 45 of the 656 samples were excluded because no water chemistry data were provided by the citizen scientists.

We used permutational analysis of variance (ANOVA) (PERMANOVA) (67) to determine if there were significant differences in relative abundances of the genus *Mycobacterium* (16S rRNA gene sequence data) or of individual mycobacterial lineages (hsp65 sequence data) across the following sample categories: household water source (municipal versus well), showerhead type, and household location (United States versus Europe).

We next sought to identify whether there were spatial (i.e., geographical) differences in the relative abundances of the genus *Mycobacterium* (16S rRNA gene sequence data) or individual mycobacterial lineages (hsp65 sequence data). As our samples were not collected randomly across the United States and Europe (sampling intensity tended to track population density [with more samples collected from regions with larger cities]), we first used hierarchical clustering employing the hclust algorithm from the R “stats” package (https://cran.r-project.org) to group these samples into regional clusters based on their geographic proximity, excluding those clusters represented by fewer than 10 samples. Using these criteria, we ended up with 21 regional clusters of samples (shown in Fig. 4 [see also Fig. S8 and S9]) and the maximum distance between sampling locations within each cluster ranged from 170 to 550 km and from 200 to 510 km (east-west distances and north-south distances, respectively). Eighty of the 656 samples did not fall into any of these 21 clusters; i.e., 80 samples were too spatially isolated to be assigned to any of the identified spatial clusters. For the 21 geographic clusters identified, we then used PERMANOVA to determine whether there were significant differences in mycobacterial abundances across the regions. Spatial clustering was included as a fixed factor in these analyses. Similar analyses were conducted to determine if there were significant differences in measured water chemistry parameters across the 21 geographic regions; those analyses were conducted to assess the degree to which the observed regional differences in mycobacterial communities were related to differences in the measured water chemistry parameters.

We used PERMANOVA to determine if there were significant differences in water chemistry across water sources (municipal versus well) and across households in the United States versus Europe. We also conducted semipartial correlations (Spearman) using the ppcor R package (68) to evaluate the correlation of water chemistry data (total chlorine, free chlorine, pH, hardness, alkalinity, and total iron, nitrite, and nitrate) with the relative abundances of *Mycobacterium* (16S rRNA gene sequence data) and individual mycobacterial lineages (hsp65 sequence data). The mean annual temperature for each household location (from the Worldclim database at worldclim.org) was also included in these analyses. Unlike standard correlations, semipartial correlations allow us to identify the variance from a given response variable that is uniquely predictable from a given predictor, controlling for all other predictors simultaneously (69). We used a heat map (heatmap.2 function in the R package gplots) to visualize our results.

Significant correlations (*P < *0.05) between the median mycobacterial abundance and prevalence estimates of pulmonary NTM disease generated from prior U.S. population-level studies of Medicare beneficiaries (50) and persons with cystic fibrosis (70) were evaluated at the state level for MAC and M. abscessus. These analyses were performed using SAS 9.4 (Cary, NC, USA).

### Data availability.

All data used in this study are publicly available in Figshare (https://figshare.com/s/02a2a13fd59618577115).
