# Supplementary figures and images for: Ecological Analyses of Mycobacteria in Showerhead Biofilms and Their Relevance to Human Health
Source: mBio. 2018 Oct 30;9(5):e01614-18. doi: 10.1128/mBio.01614-18 (PMC6212831; doi:10.1128/mBio.01614-18)

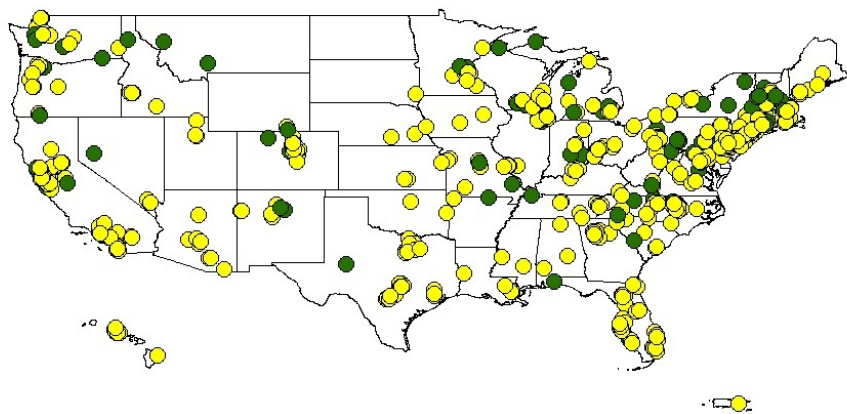

**■ Municipal ■ Well**

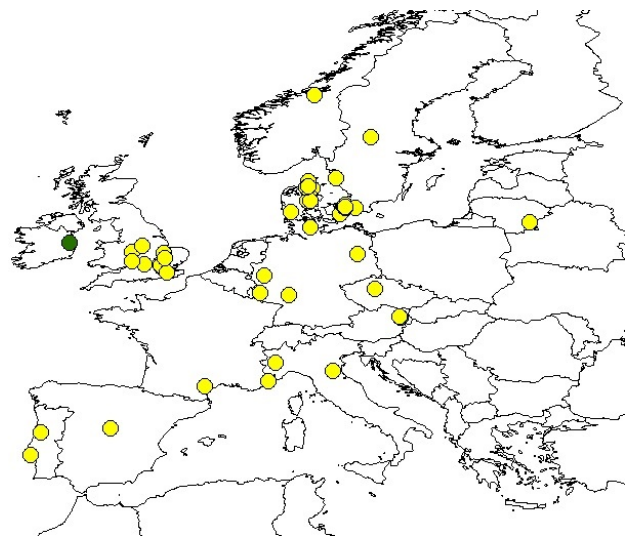

Supplement: FIG S1 [file mbo005184117sf1.pdf]

Abundance (%)

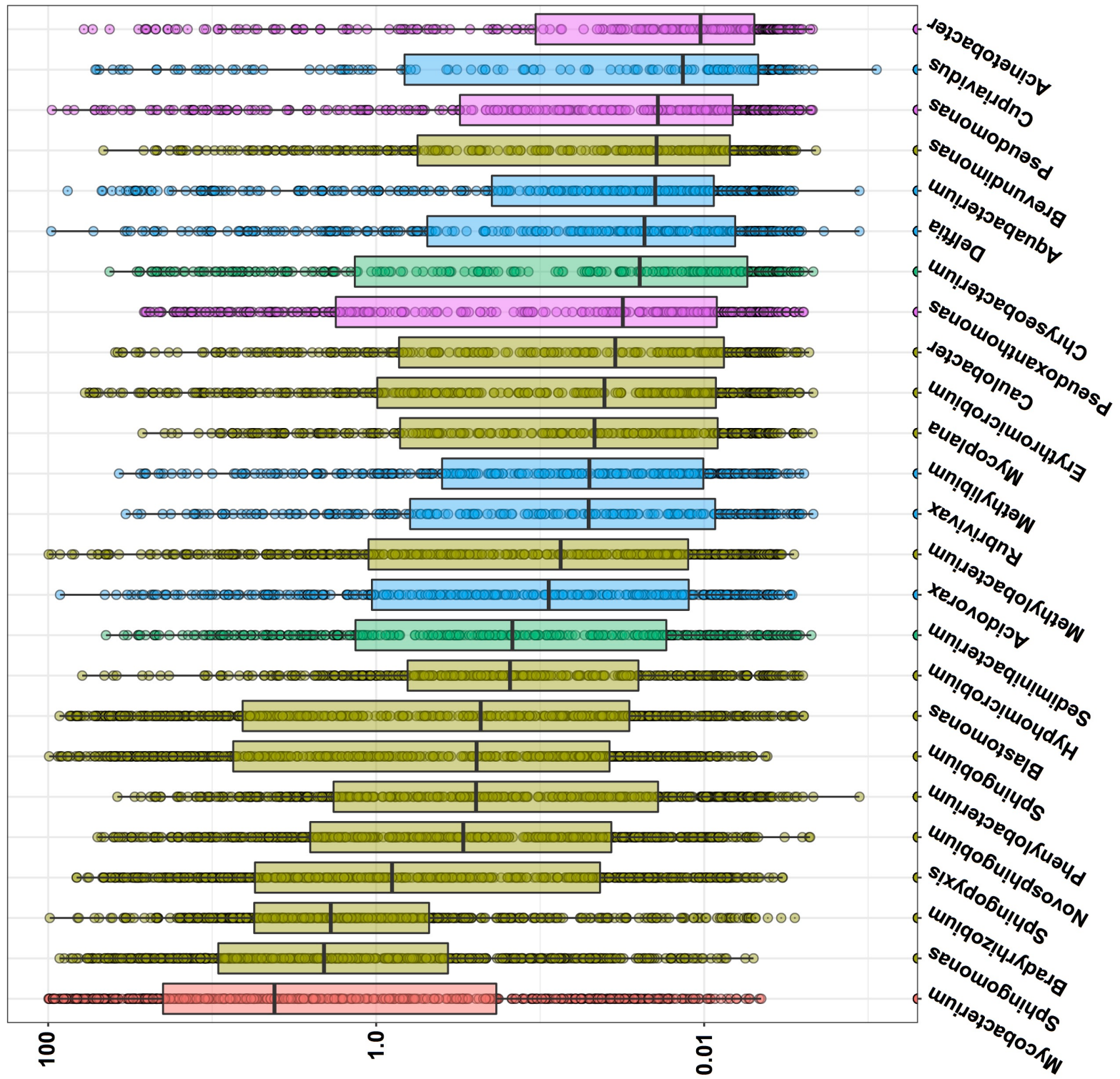

Supplement: FIG S2 [file mbo005184117sf2.pdf]

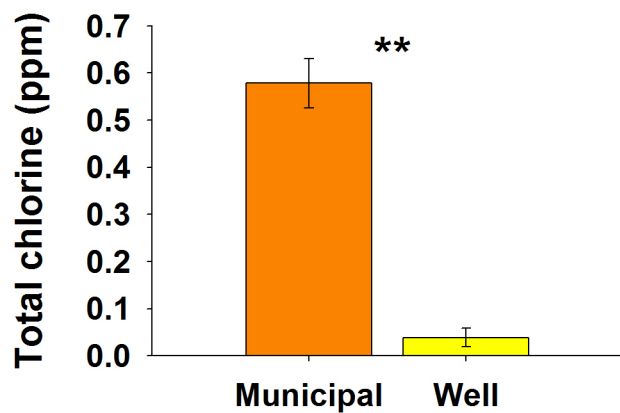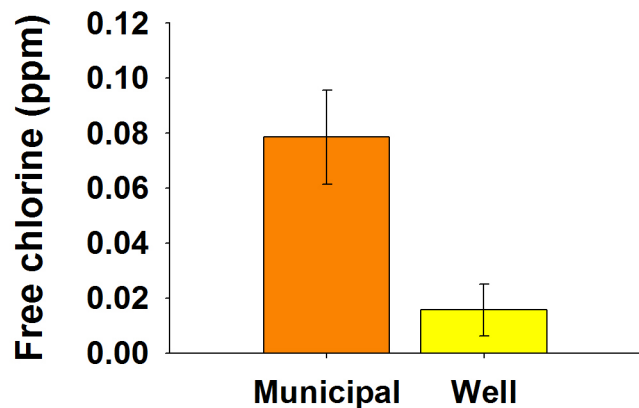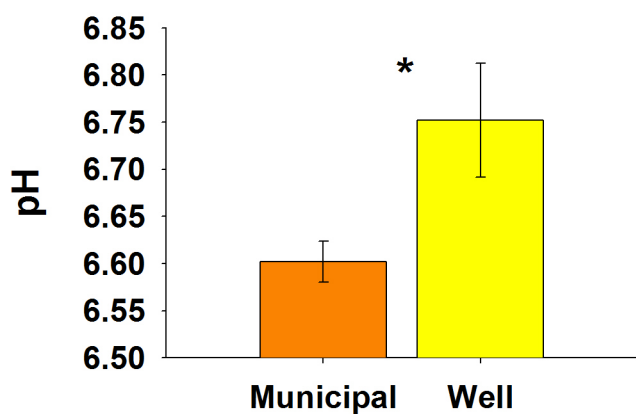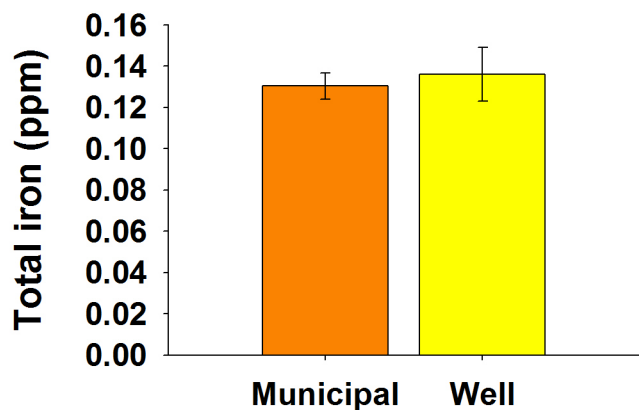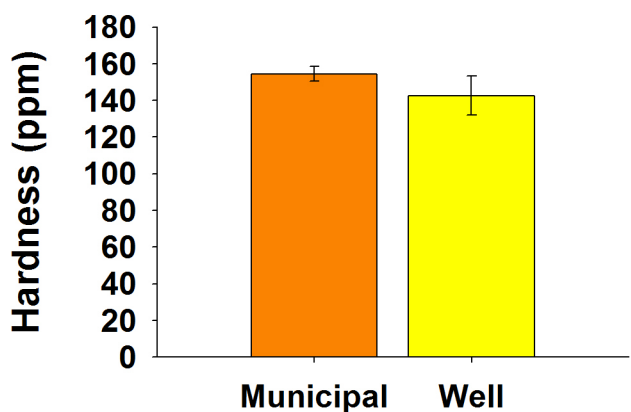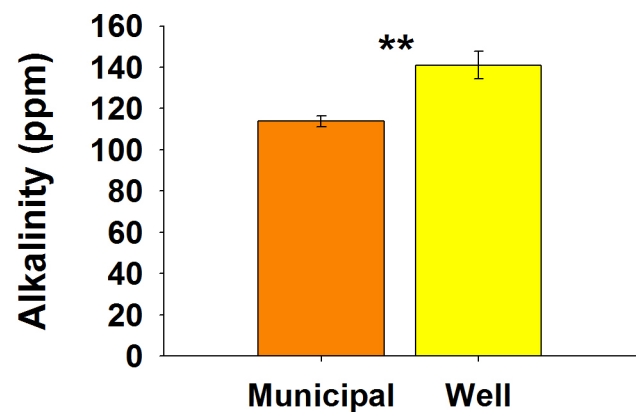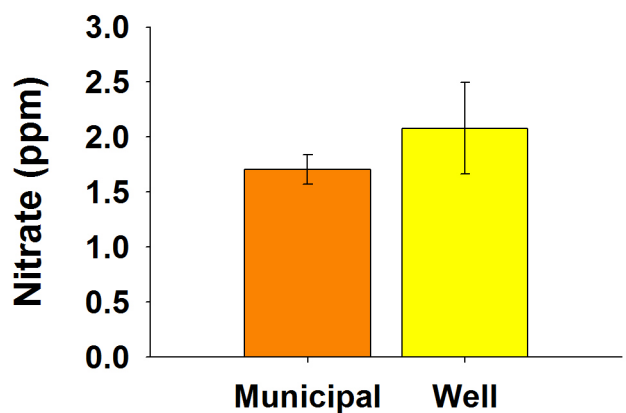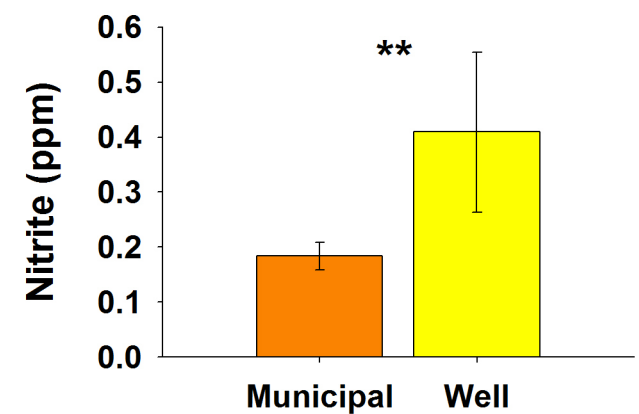

Supplement: FIG S3 [file mbo005184117sf3.pdf]

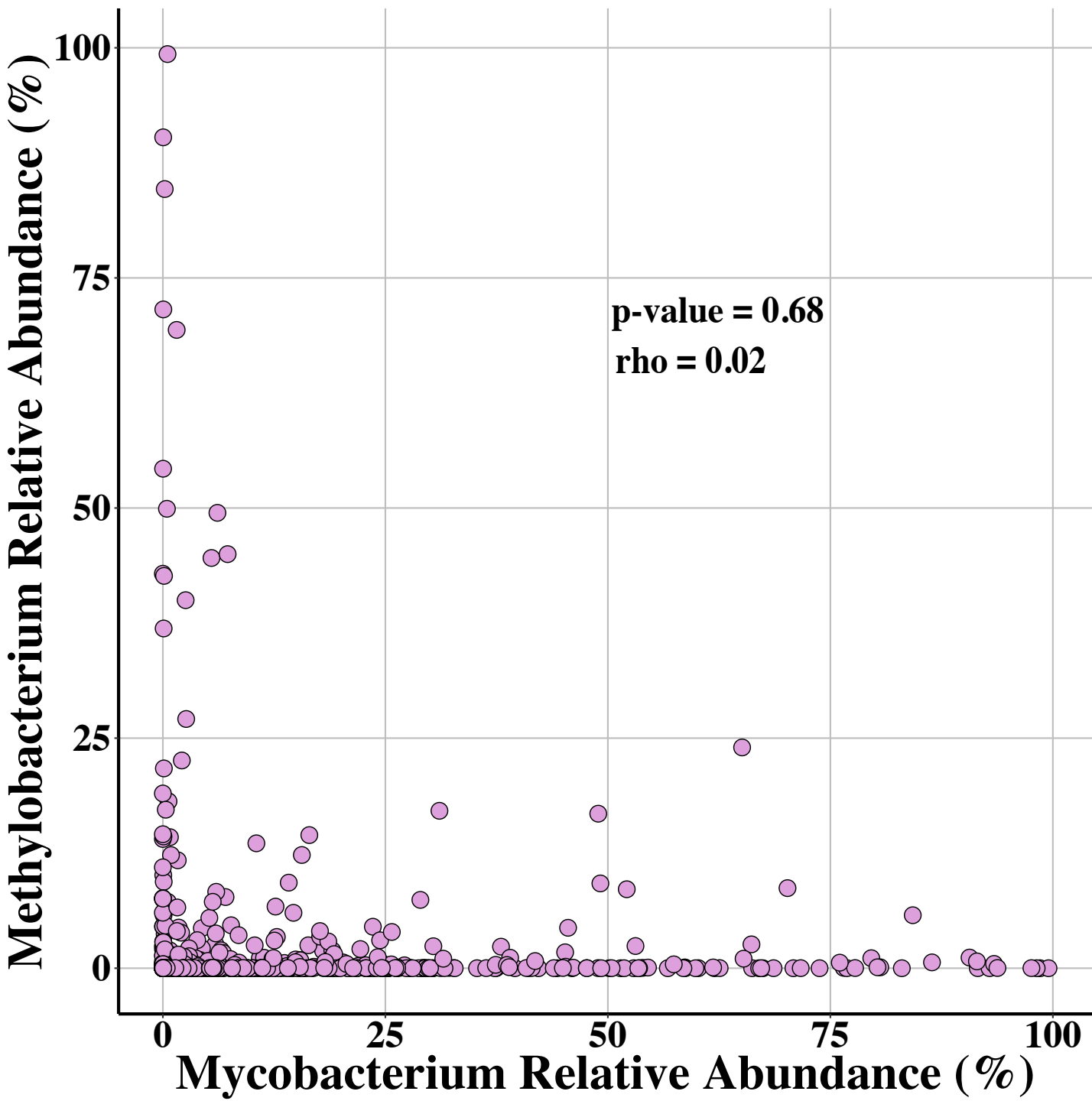

Supplement: FIG S4 [file mbo005184117sf4.pdf]

Relative abundance of *Mycobacterium* (%)

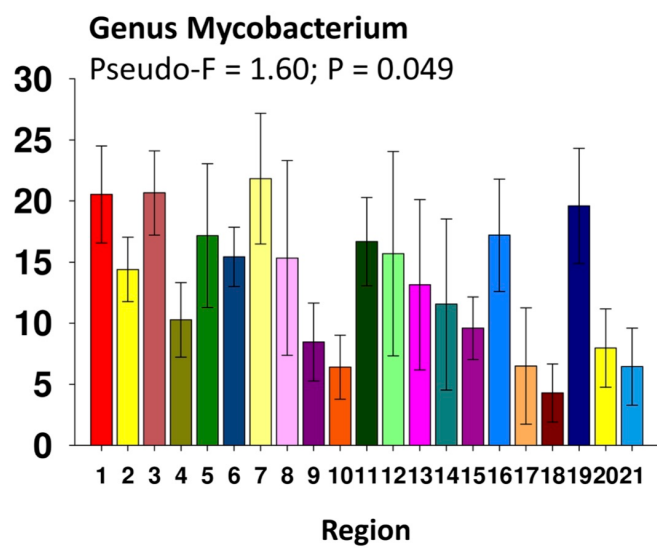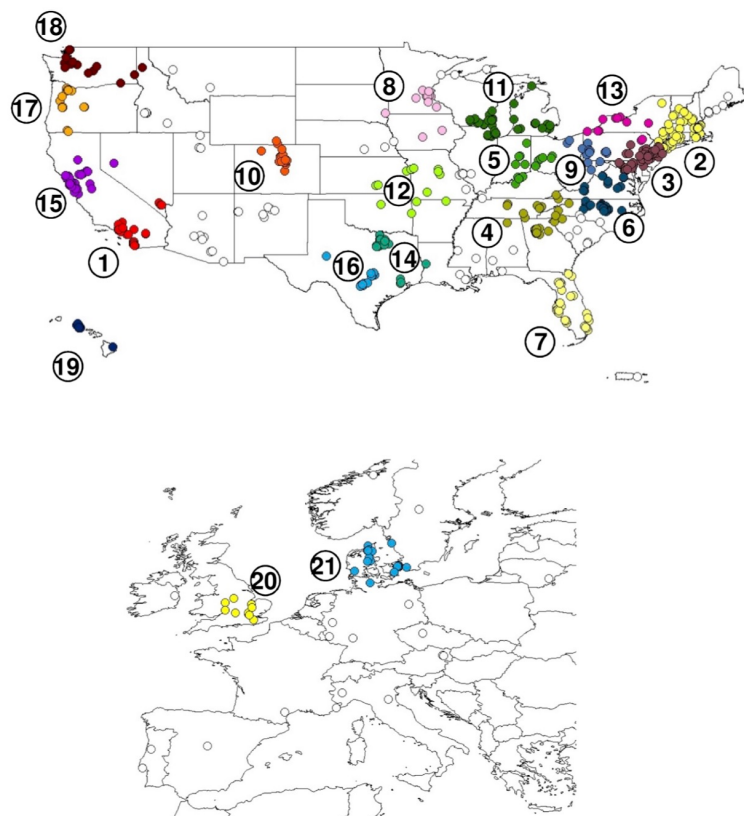

Supplement: FIG S5 [file mbo005184117sf5.pdf]

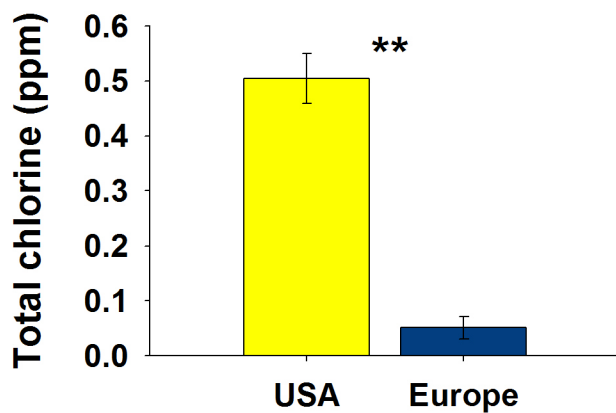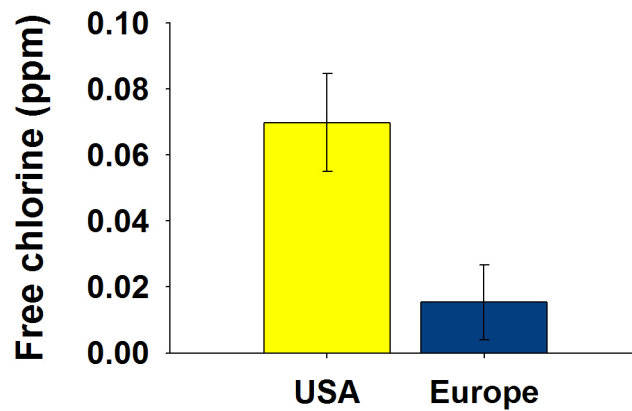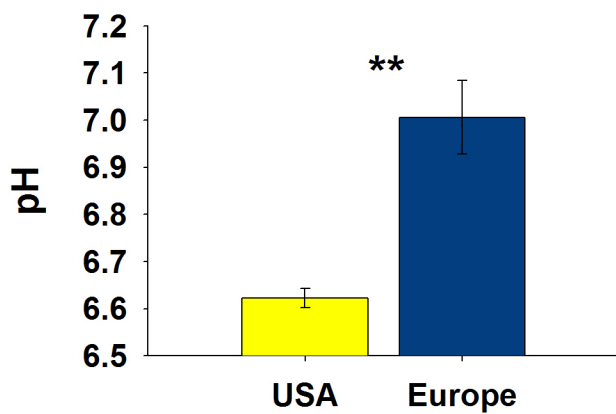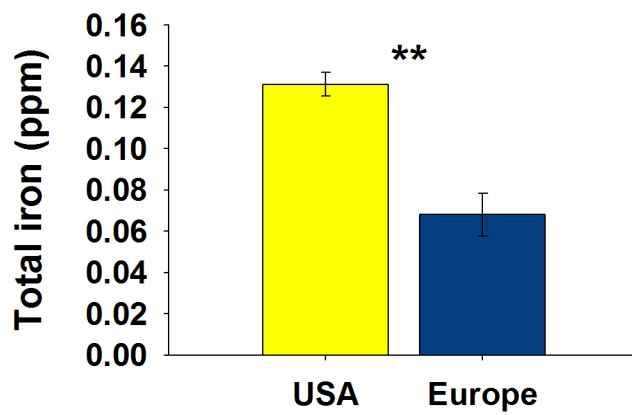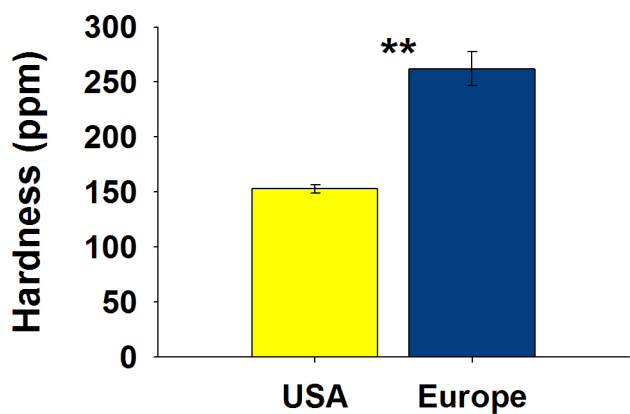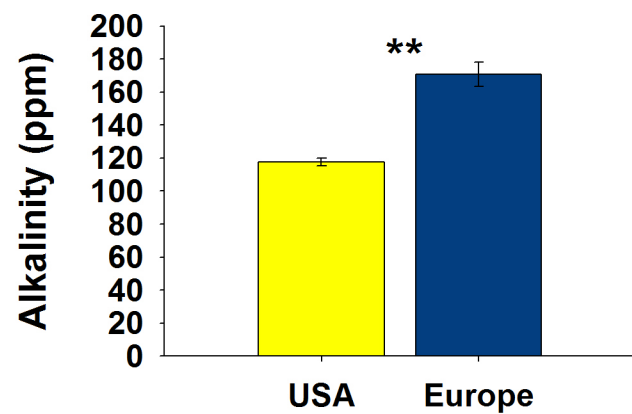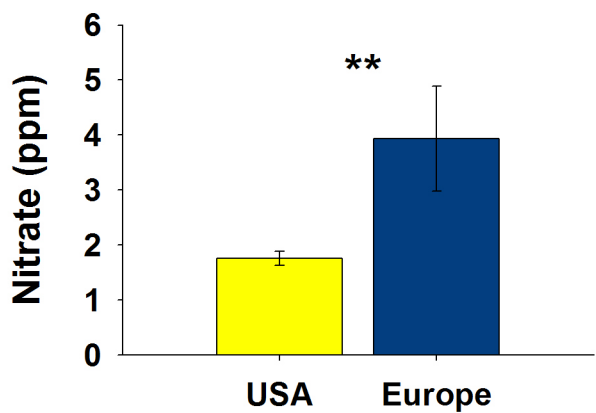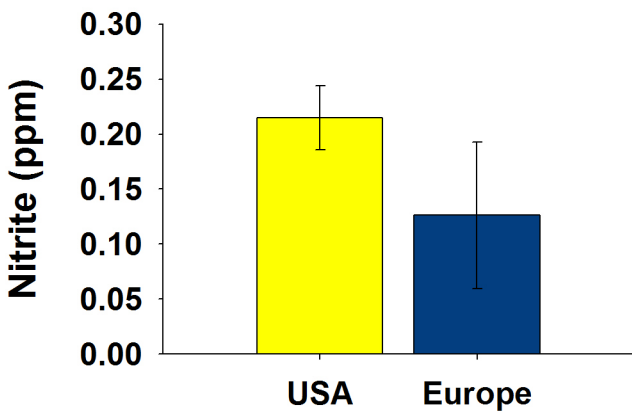

Supplement: FIG S6 [file mbo005184117sf6.pdf]

- Culture Dependent and Independent
- Culture Independent Only
- Culture Dependent Only
- Other

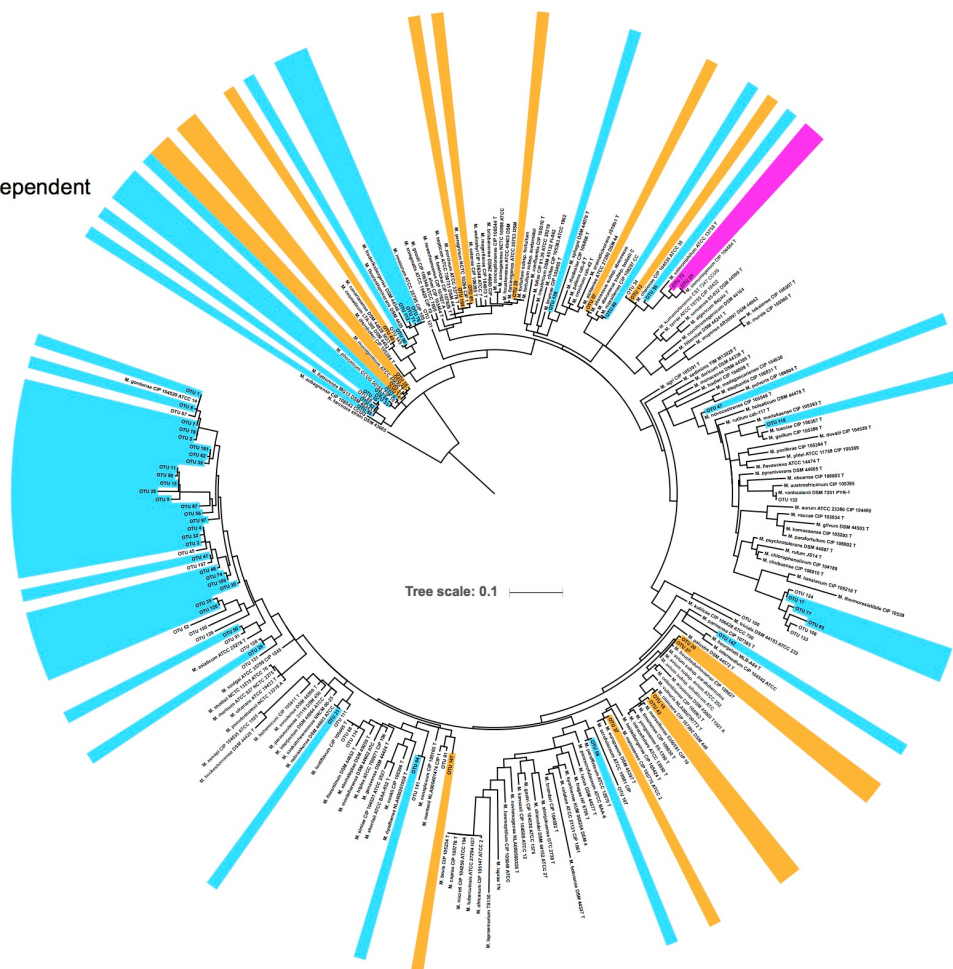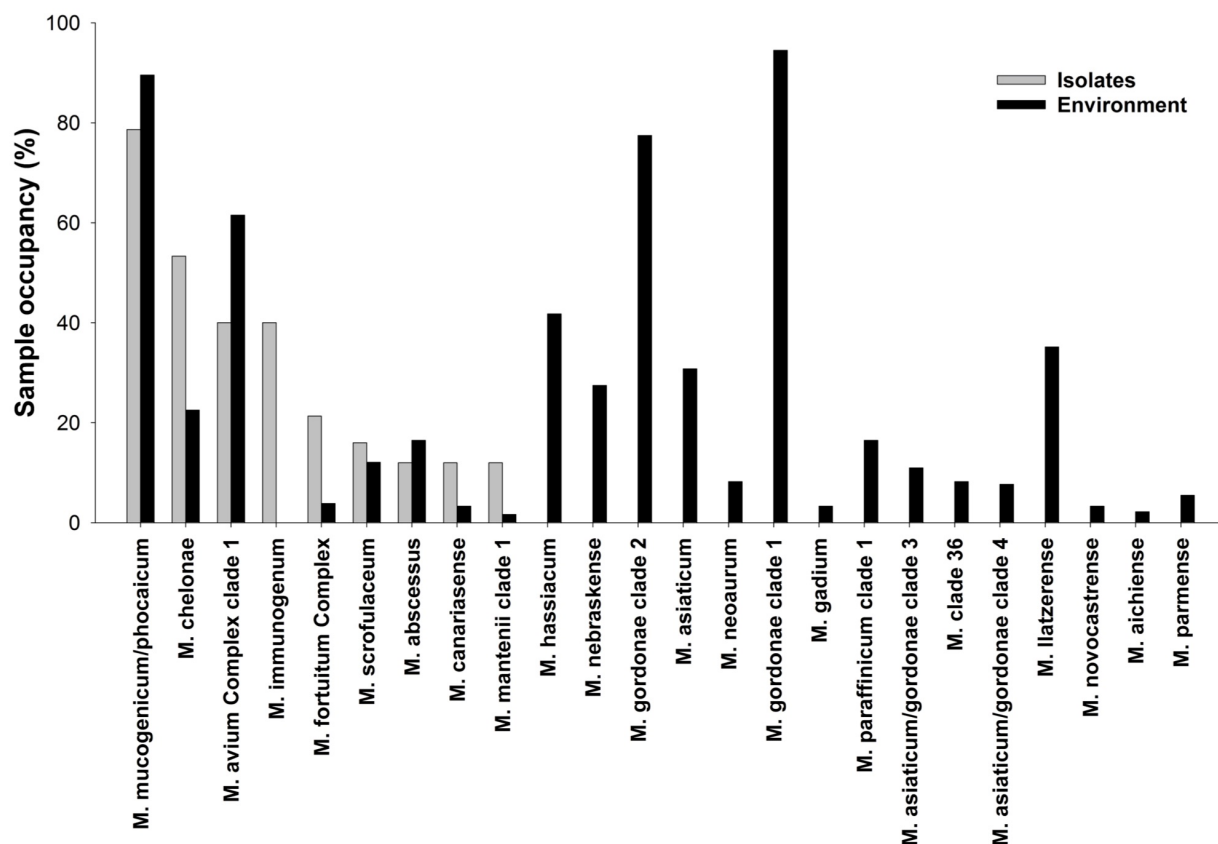

Supplement: FIG S7 [file mbo005184117sf7.pdf]

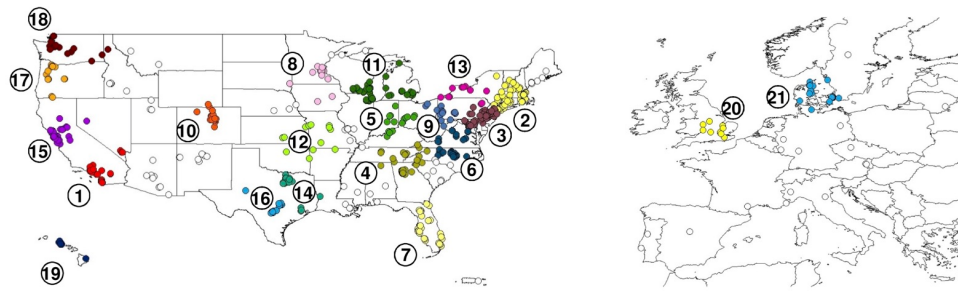

Relative abundance of *Mycobacterium* (%)

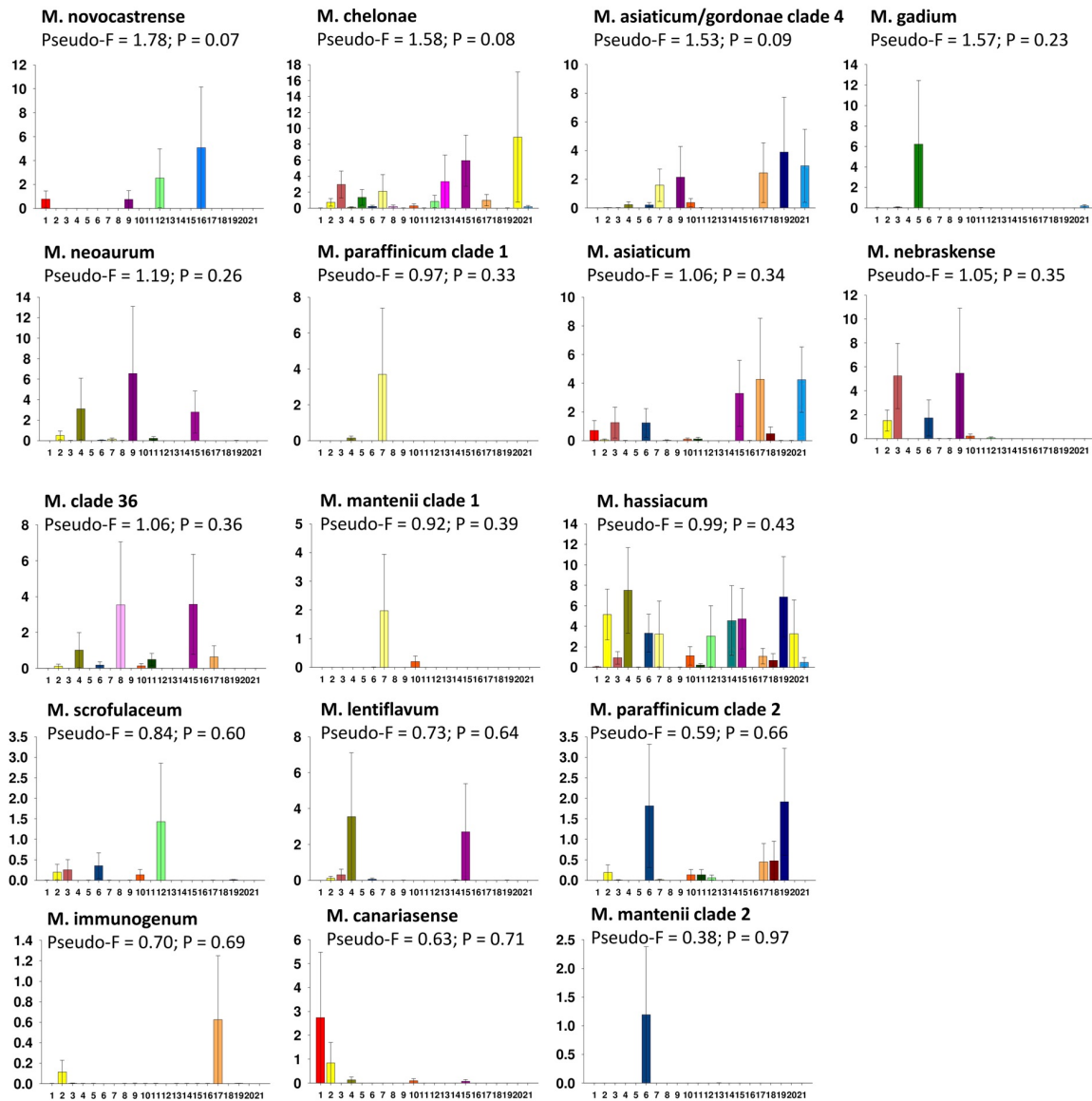

Region

Supplement: FIG S9 [file mbo005184117sf9.pdf]
