# Supplementary material for: Ecological Analyses of Mycobacteria in Showerhead Biofilms and Their Relevance to Human Health
Source: mBio. 2018 Oct 30;9(5):e01614-18. doi: 10.1128/mBio.01614-18 (PMC6212831; doi:10.1128/mBio.01614-18)
Supplement: FIG S8 [file mbo005184117sf8.pdf]

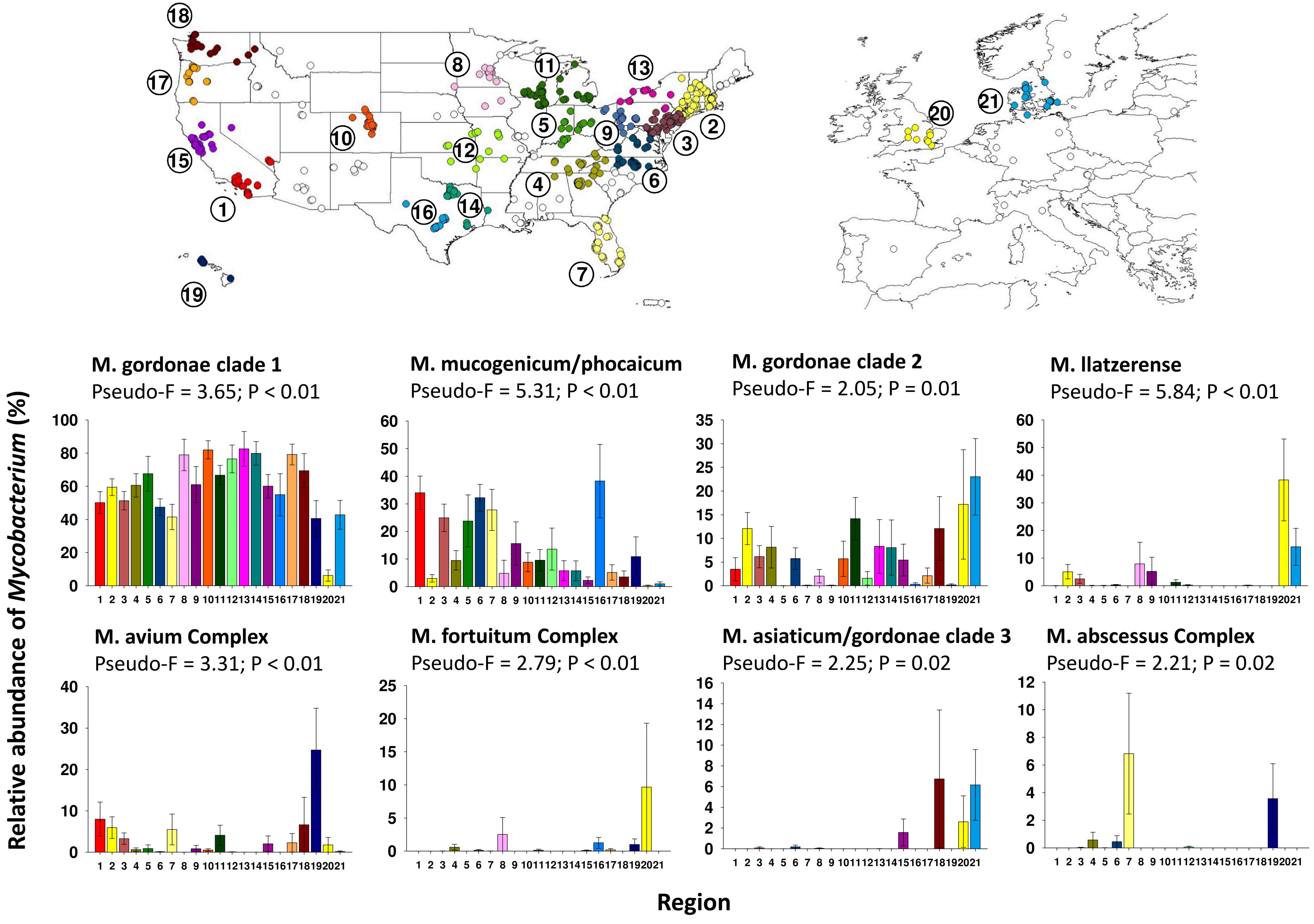
18
17
15
1
19
10
8
12
16
14
11
5
4
7
9
13
2
3
6
20
21
M. gordonae clade 1
Pseudo-F = 3.65; P < 0.01
M. mucogenicum/phocaicum
Pseudo-F = 5.31; P < 0.01
M. gordonae clade 2
Pseudo-F = 2.05; P = 0.01
M. llatzerense
Pseudo-F = 5.84; P < 0.01
M. avium Complex
Pseudo-F = 3.31; P < 0.01
M. fortuitum Complex
Pseudo-F = 2.79; P < 0.01
M. asiaticum/gordonae clade 3
Pseudo-F = 2.25; P = 0.02
M. abscessus Complex
Pseudo-F = 2.21; P = 0.02
Relative abundance of *Mycobacterium* (%)
Region
