# Supplementary material for: Ecological Analyses of Mycobacteria in Showerhead Biofilms and Their Relevance to Human Health
Source: mBio. 2018 Oct 30;9(5):e01614-18. doi: 10.1128/mBio.01614-18 (PMC6212831; doi:10.1128/mBio.01614-18)
Supplement: FIG S10 [file mbo005184117sf10.pdf]

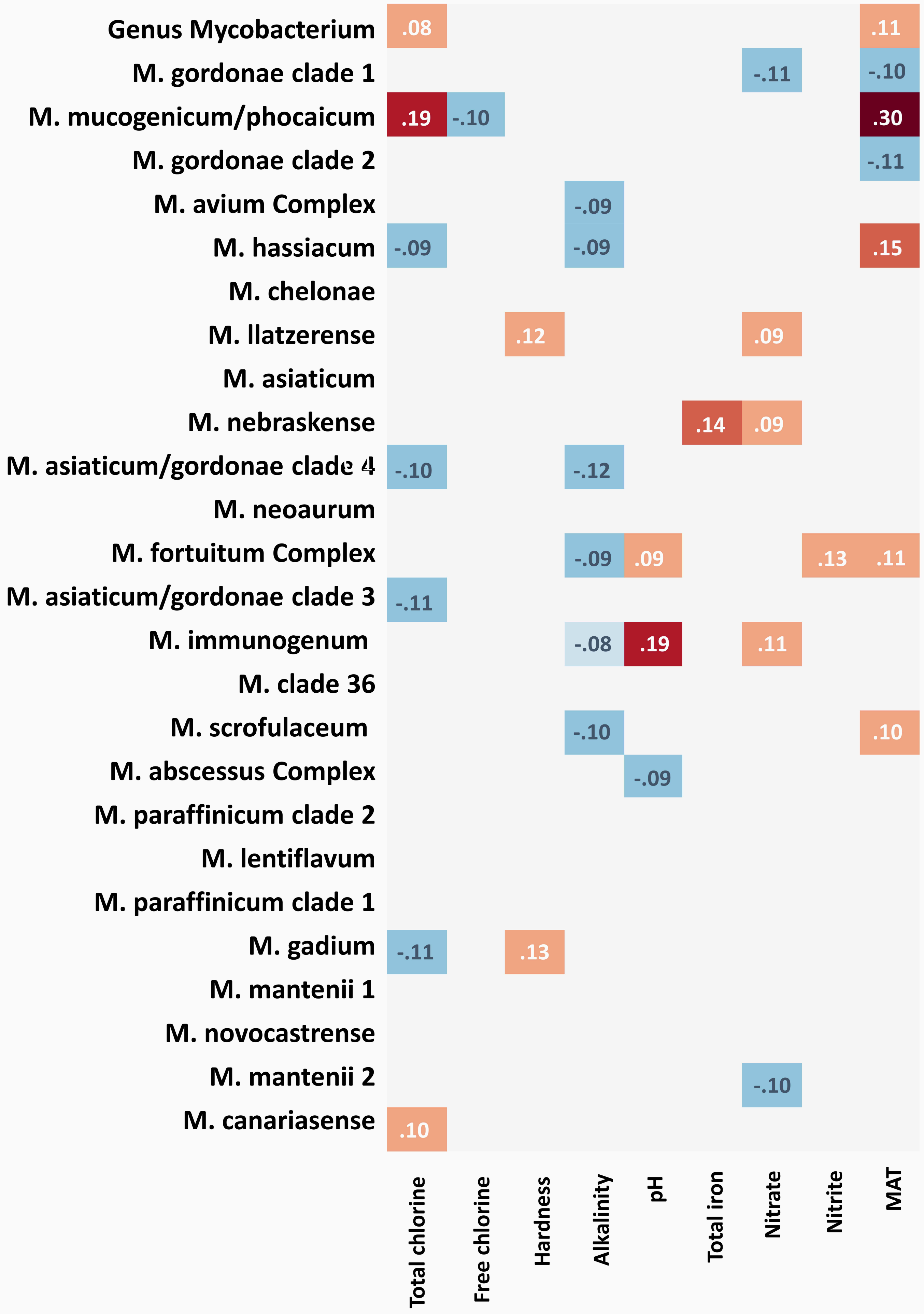
Genus Mycobacterium
.08
.11
M. gordonae clade 1
-.11
-.10
M. mucogenicum/phocaicum
.19
-.10
.30
M. gordonae clade 2
-.11
M. avium Complex
-.09
M. hassiacum
-.09
-.09
.15
M. chelonae
M. llatzerense
.12
.09
M. asiaticum
M. nebraskense
.14
.09
M. asiaticum/gordonae clade 4
-.10
-.12
M. neoaurum
M. fortuitum Complex
-.09
.09
.13
.11
M. asiaticum/gordonae clade 3
-.11
M. immunogenum
-.08
.19
.11
M. clade 36
M. scrofulaceum
-.10
.10
M. abscessus Complex
-.09
M. paraffinicum clade 2
M. lentiflavum
M. paraffinicum clade 1
M. gadium
-.11
.13
M. mantenii 1
M. novocastrense
M. mantenii 2
-.10
M. canariasense
.10
Total chlorine
Free chlorine
Hardness
Alkalinity
pH
Total iron
Nitrate
Nitrite
MAT
